# TEG-based transfusion protocol is associated with decreased blood product use without increased risk of hemoperitoneum

**DOI:** 10.1097/HC9.0000000000000292

**Published:** 2023-10-27

**Authors:** Brittany Bromfield, Roberto Tellez, Dempsey L. Hughes, Rebecca Brown, Margaret Andrzejewski, Aditi Bawa, Fei-Pi Lin, Mitchell Tublin, Darrell Triulzi, Armando Ganoza, Andres Duarte-Rojo

**Affiliations:** 1 Department of Internal Medicine, University of Pittsburgh, Pittsburgh, Pennsylvania, USA; 2 Division of Gastroenterology and Hepatology, Northwestern University, Chicago, Illinois, USA; 3 University of Pittsburgh School of Medicine, Pennsylvania, USA; 4 Division of Gastroenterology, Hepatology and Nutrition, University of Pittsburgh, Pittsburgh, Pennsylvania, USA; 5 Department of Radiology, University of Pittsburgh, Pittsburgh, Pennsylvania, USA; 6 Department of Pathology, University of Pittsburgh, Pittsburgh, Pennsylvania, USA; 7 Department of Surgery, University of Pittsburgh, Pittsburgh, Pennsylvania, USA

## Abstract

**Background:**

Thromboelastography (TEG) informs the need for blood product transfusions to prevent procedural bleeding complications in patients with cirrhosis. We aimed to evaluate the impact of using a TEG-based transfusion protocol on blood product utilization before paracentesis and the post-paracentesis hemoperitoneum (PPH) incidence.

**Methods:**

We conducted an ambispective analysis of patients with cirrhosis who underwent paracentesis from 2017 to 2021. In May 2019, we enacted a TEG-based transfusion protocol to guide pre-paracentesis blood product use. Patients with platelets < 20,000 or international normalized ratio ≥ 4 underwent TEG and received blood products if *r* value > 10 min or MA <30 mm. Patients were divided into pre-TEG and post-TEG protocol cohorts based on the date of paracentesis. Pre-paracentesis blood product transfusions in the form of platelets, fresh frozen plasma, and cryoprecipitates were recorded. PPH was defined as a decrease in hemoglobin of ≥1 g and the presence of blood on diagnostic imaging and/or the need for therapeutic intervention.

**Results:**

A total of 483 patients underwent 1281 paracenteses. The main etiologies of cirrhosis were alcohol (43%) and NASH (25%), and the mean MELD-sodium was 22±6. Pre-TEG and post-TEG protocol cohort sizes were similar: 253 patients and 607 paracenteses versus 230 patients and 674 paracenteses. After TEG-protocol implementation, blood product transfusions decreased significantly (228 vs. 49 products, *p*<0.001) with associated cost savings. One patient in each cohort developed PPH.

**Conclusion:**

Implementation of a pre-paracentesis TEG-based transfusion protocol for patients with cirrhosis successfully resulted in decreased blood product use with no associated increase in incidence of PPH.

## Graphical abstract

**Figure:**
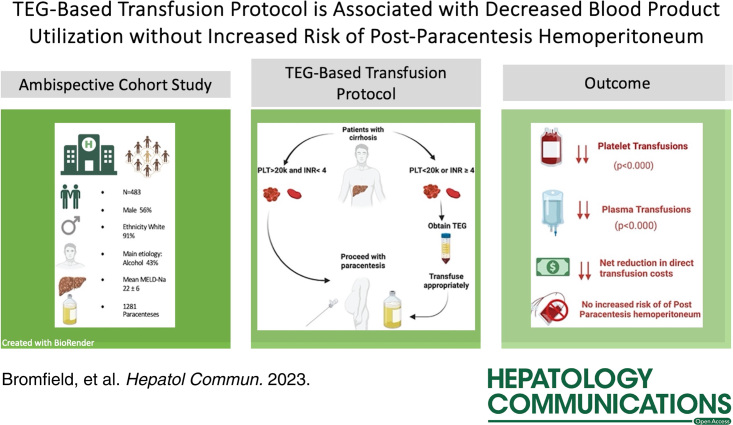

## INTRODUCTION

The risk of procedural bleeding in patients with cirrhosis can be difficult to determine due to concurrent thrombocytopenia and coagulopathy. Historically, patients with advanced liver disease were presumed to have a higher risk of periprocedural bleeding based on abnormal levels of standard laboratory parameters, including low platelets (PLT) and elevated INR. However, further studies have demonstrated that cirrhosis constitutes a state of “rebalanced” coagulation profile. Contributing factors to this distinct coagulation profile include diminished hepatic synthesis of clotting factors and reduced PLT secondary to splenic sequestration, which are offset by a reduction of anticoagulant proteins and preserved generation of thrombin.^1–3^ Consequently, accurately determining the associated risk of procedural bleeding in such a complex state is beyond the means of conventional laboratory markers like INR. Nevertheless, these conventional parameters have often driven the use of blood transfusions despite limited evidence of clinical benefit.^4,5^

Thromboelastography (TEG) is a point-of-care test that gives a global assessment of hemostatic function (clot formation and lysis) reflected in real time.^6^ Per randomized controlled trials, the use of TEG in patients with cirrhosis has been associated with the safe and effective utilization of blood products.^7–9^ More specifically, these trials have demonstrated considerably lower use of blood products when based on TEG values as opposed to conventional parameters (PLT, INR). We aimed to evaluate the impact of a TEG-based transfusion protocol on blood product utilization before paracentesis and the incidence of post-paracentesis hemoperitoneum (PPH).

## METHODS

### Patient selection

In this ambispective analysis, we evaluated adult patients with cirrhosis who underwent paracentesis at the University of Pittsburgh Medical Center from 2017 to 2021. Data were retrieved from the University of Pittsburgh Medical Center’s secure electronic medical record. We included patients ≥ 18 years old with established cirrhosis. The diagnosis of cirrhosis was based on clinical presentation, diagnostic imaging, or prior liver biopsy. Exclusion criteria included patients under the age of 18 years and patients with any evidence of active bleeding at the time of paracentesis. All patients and/or their medical decision-makers consented to paracentesis. Paracentesis was performed in either an inpatient or outpatient setting, and all were performed under ultrasound guidance per institutional protocol.

### Study design

Patients were divided into pre-TEG and post-TEG protocol cohorts based on the date of paracentesis with respect to the time of protocol implementation (June–July 2019). All patients were followed clinically for the next 48 hours for the onset of PPH, including daily hemoglobin checks. PPH was defined as a drop in hemoglobin ≥ 1 gram plus one or more of the following: the presence of i.p. bleeding on diagnostic abdominal imaging or the need for therapeutic intervention (interventional radiology or surgery). Our primary outcome was the difference in the total number of blood products transfused (PLT, plasma/cryoprecipitate) between the pre-TEG and post-TEG protocol cohorts. Secondary outcomes were the onset of PPH within 48 hours after paracentesis and direct health care costs attributed to blood product use and TEG processing in the 2 cohorts, respectively.

### Thromboelastography

**FIGURE 1:**
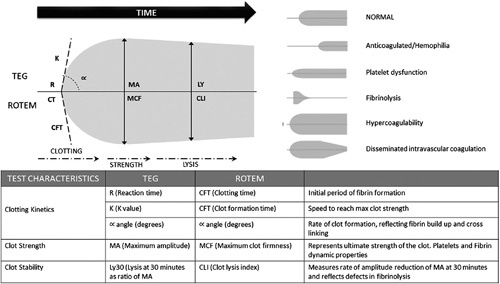
TEG schematic characteristics and interpretation. TEG is a viscoelastic hemostatic assay that measures the global properties of whole blood clot formation. It shows the interaction of platelets with the coagulation cascade, including aggregation, clot strengthening, fibrin cross-linking, and fibrinolysis. TEG provides a global assessment of hemostatic function. Reproduced with permission from Garcia-Saenz-de-Sicilia M, et al., 2022.^10^ Abbreviations: CFT, cloth formation time; CLI, clot lysis index; K, K value; Ly30, lysis 30; MA, maximum amplitude; MCF, maximum clot firmness; R, reaction time; TEG, thromboelastography.

TEG is a point-of-care, global, hemostatic tool that evaluates the dynamics of clot formation. It provides real-time data on clot strength and stability between soluble clotting factors and inhibitors and PLT (Figure 1). In this study, the following parameters of TEG were used:^11^

- *r* value: A prolonged *r* value (>10 min) reflects a quantitative or qualitative deficiency of coagulation factors, which may be corrected by plasma transfusion, prothrombin complex, or anticoagulant reversal.
- Maximum amplitude (MA): A low MA (<30 mm) indicates a quantitative or functional deficiency of PLT and could be corrected by platelet concentrate transfusion.

### TEG-based transfusion protocol

**FIGURE 2:**
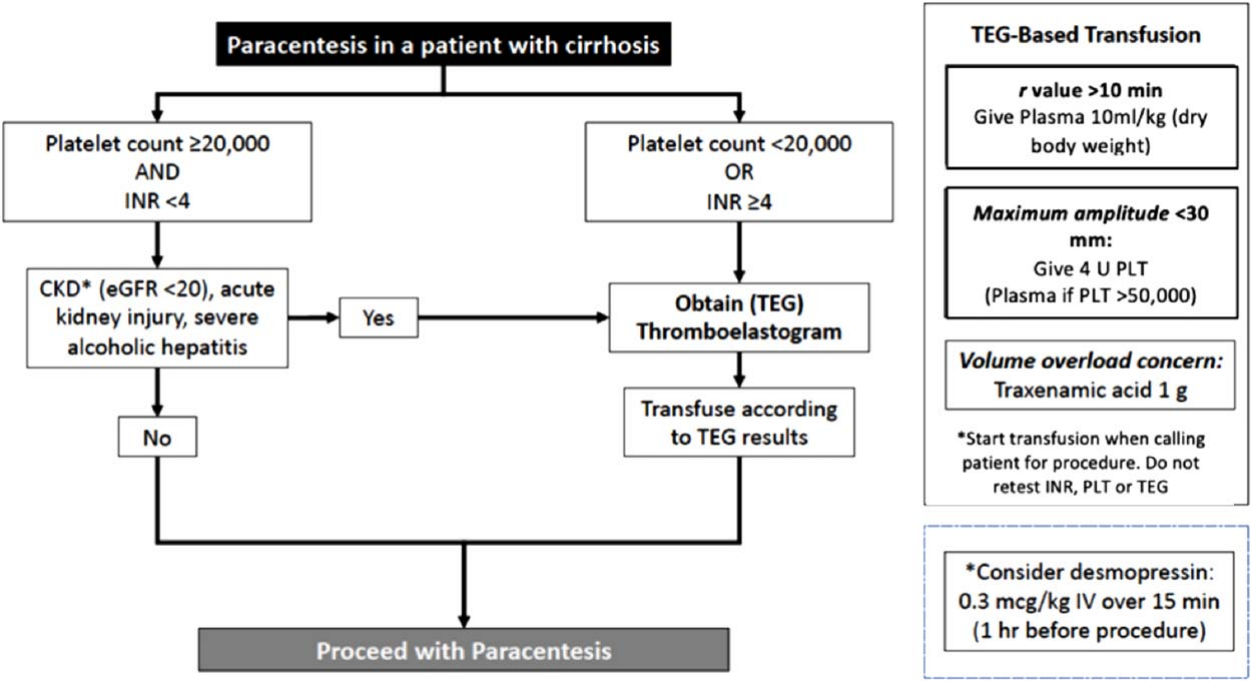
Thromboelastography (TEG)-based transfusion protocol algorithm. Patients with cirrhosis undergoing paracentesis are evaluated based on their coagulation parameters. The presence of low platelets and/or elevated INR determines whether a TEG is ordered and whether transfusion of blood products is warranted. The protocol also takes into account renal dysfunction as well as acute inflammatory states such as severe alcohol-associated hepatitis. Abbreviations: PLT, platelet; TEG, thromboelastography.

In May 2019, we enacted a TEG-based transfusion protocol utilizing an evidence-based algorithm to guide blood product transfusion (Figure 2). The algorithm consisted of laboratory values and clinical comorbidities traditionally associated with the risk of periprocedural bleeding.^9,12–14^ Patients meeting clinical criteria for higher bleeding risk underwent TEG to determine if any preprocedural blood product transfusion was warranted. Recommendations, however, did not apply to patients on anticoagulation or antiplatelet therapy other than aspirin. There was also a consideration for desmopressin and tranexamic acid for conditions associated with platelet or coagulation dysfunction.^15^ The protocol also instructed the time of blood product transfusion and explicitly advised against repeat laboratory testing (ie, to confirm a corrected parameter) before the procedure. The thresholds for PLT, INR, and TEG parameters were determined by a multidisciplinary group of physicians in hepatology, interventional radiology, and transfusion medicine. Multiple interdisciplinary meetings were performed to review existing relevant literature on the topic and develop the TEG-based protocol. These faculty meetings occurred from January to April 2019.

Once our TEG-based protocol was finalized, an implementation phase (May–June of 2019) included dissemination of the protocol among providers in charge of paracentesis and education on the interpretation of TEG results. We accomplished this by posting copies of the protocol in pertinent clinical working areas (including the dedicated area for outpatient paracentesis and the hepatology inpatient service), training nurses and advanced practice providers involved in the care of patients undergoing paracentesis, and frequently reminding protocol workflow during hepatology faculty meetings as well as in internal grand rounds or other educational efforts for trainees. As a key component of implementation, the authors gave special emphasis on the education of providers placing the orders for paracentesis so that they could identify when to request the TEG and the need for transfusion and on the providers performing the paracentesis so that they could confirm the transfusional intervention was properly executed before paracentesis. The senior author (Andres Duarte-Rojo) made himself available to any provider in need of advice, and our transfusion medicine expert (Darrell Triulz) was consulted when in doubt. In consideration of a suboptimal uptake of the TEG-based protocol during the initial phase of implementation, we eliminated subjects who underwent paracentesis during May and June of 2019. The TEG-based protocol was registered as a Quality Improvement Project at the corresponding UPMC website and approved by overseeing authorities.

### Data collection

We collected clinical and demographic data, including age, sex, ethnicity, BMI, etiology, cirrhosis-related complications, and other medical comorbidities (diabetes mellitus, kidney disease, coronary artery disease, and aspirin usage). All patients had pre-paracentesis hemoglobin, serum electrolytes, renal function, liver function panel, and coagulation studies, as well as post-paracentesis hemoglobin and serum creatinine. Procedural data were also collected, including paracentesis setting (ie, inpatient vs. outpatient), ascites volume drained during paracentesis, and dose of albumin given after the procedure. Lastly, any preprocedural transfusion of PLT, plasma, and cryoprecipitate was also recorded. Charts were independently reviewed by 7 investigators (Brittany Bromfield, Roberto Tellez, Dempsey L. Hughes, Andres Duarte-Rojo, Fei-Pi Lin, Aditi Bawa, and Rebecca Brown). Direct costs of blood products transfused and the direct cost of TEG assay performance were obtained from 2021 internal operations cost data from the Division of Laboratory Medicine, Department of Pathology at the University of Pittsburgh. Costs were in US dollars.

### Statistical analysis

Demographics and clinical characteristics of the cohort were described using frequency and percentages for categorical variables, while continuous variables were listed as mean ± SD or median (25th–75th percentiles). Chi-square, Student’s t, or Wilcoxon tests were used for comparisons, as appropriate. Statistical significance was defined as *p* < 0.05. All analyses were performed using the Stata 16.1 software (StataCorp, College Station, TX). This study was approved by the University of Pittsburgh’s Institutional Review Board. The manuscript was reviewed and approved by all authors prior to submission.

## RESULTS

### Demographic and clinical characteristics

We identified 483 patients with cirrhosis who underwent a total of 1281 paracenteses during the study period. The pre-TEG protocol cohort consisted of a total of 253 patients with cirrhosis who underwent 607 paracenteses from September 2017 to April 2019. The post-TEG protocol cohort consisted of a total of 230 patients with cirrhosis who underwent 674 paracenteses from July 2019 to September 2021 (Table 1). When comparing patients before and after the TEG-based protocol, the 2 cohorts were similar with respect to demographics and clinical characteristics. The majority of patients in each cohort were male and had similar mean ages. The most common cause of cirrhosis was alcohol, followed by NASH. There were no differences in the severity of liver disease in terms of MELD-sodium 22±6 before the TEG-based protocol and 23±6 after the TEG-based protocol, and the type/frequency of decompensating events. Aspirin utilization was similar for the 2 periods as well. The baseline characteristics of the cohort when accounting for paracenteses episodes are shown in Supplemental Table, http://links.lww.com/HC9/A599. With respect to paracentesis equipment, 184 (14%) paracenteses were performed for diagnostic purposes with a spinal needle, whereas 1097 (86%) were performed with a 5- or 6-French nonlocking catheter.

**TABLE 1:** Baseline demographic and clinical characteristics of the entire cohort

	Total N =483	Period 1 N=253	Period 2 N=230	*P*
Age				
Mean	57±13	57±13	57±12	1.0
Sex, n (%)				
Male	272 (56)	150 (59)	122 (53)	—
Female	211 (44)	103 (41)	108 (47)	0.17
Race, n (%)				
White	440 (91)	231 (91)	209 (91)	0.75
Black	14 (3)	8 (3)	6 (3)	—
Asian	2 (0)	0 (0)	2 (1)	—
Other	60 (6)	14 (6)	46 (5)	—
Etiologies of cirrhosis, n (%)				
NASH	122 (25)	58 (23)	64 (28)	0.51
Alcohol	206 (43)	107 (42)	99 (43)	—
HCV	50 (10)	26 (10)	24 (10)	—
Other	105 (22)	62 (25)	40 (19)	—
Comorbidities, n (%)				
Diabetes mellitus	163 (34)	91 (36)	72 (31)	0.34
Coronary artery disease	81 (17)	46 (18)	35 (15)	0.38
Chronic kidney disease	133 (28)	69 (27)	64 (28)	0.89
Dialysis	37 (8)	24 (9)	16 (6)	0.11
MELD-NA Score	22±6	22±6	23±6	0.65
Cirrhosis-related complications, n (%)				
HE	303 (63)	165 (65)	138 (60)	0.24
Esophageal varices	306 (63)	159 (63)	147 (64)	0.17
Variceal bleeding	77 (16)	35 (14)	42 (18)	0.18
HCC	44 (9)	19 (8)	25 (11)	0.25
Aspirin	112 (23)	59 (23)	53 (23)	0.94
Low molecular weight heparin prophylaxis	98 (20)	51 (20)	47 (20)	0.94
Paracentesis setting, n (%)				
Inpatient	392 (81)	199 (79)	193 (84)	0.14
Outpatient	91 (19)	54 (21)	37 (16)	—

### Impact of the implementation of TEG protocol on blood product utilization

Following the implementation of the TEG-based protocol, a total of 104 TEGs were performed before paracentesis, which corresponded to 15% of paracentesis performed. PLT, INR, and TEG results are shown in Table 2. Although during period 2 (post-TEG protocol) blood PLT were statistically higher (“paracentesis-based” analysis only), the difference was not considered clinically meaningful. Out of the subjects who underwent a TEG, 11% (n = 11) had abnormal values according to the protocol. Specifically, 8 patients had MA < 30 mm and 3 patients had an *r* value > 10 min. Following the implementation of the TEG-based protocol, there was a significant decrease in the utilization of blood products (Table 2). The most substantial reduction was observed in PLT transfusions between the 2 cohorts, with 90 U in the pre-TEG cohort compared to 15 U in the post-TEG cohort. A significant decrease in plasma-derived product utilization was also observed, with 138 U in the pre-TEG cohort compared to 26 U in the post-TEG cohort. These reductions came at the expense of more cryoprecipitate transfusions (0 in pre-TEG vs. 8 in post-TEG cohort). No patients in the post-TEG cohort received desmopressin or tranexamic acid.

**TABLE 2:** Hemostatic tests and blood products transfused in each cohort per paracentesis procedures and per patients

	Paracentesis-Based (Event) Analysis			
	Total N = 1281	Pre-TEG Protocol N = 607	Post-TEG Protocol N = 674	*p*
Platelet count (x10^9^/L)	102 (63–154)	98 (61–153)	109 (66–154)	0.03
INR	1.6 (1.3–2.0)	1.5 (1.3–1.9)	1.6 (1.3–2.0)	0.05
TEG				
MA (mm)	—	—	44 (31–48)	N/A
R (min)	—	—	5 (4–7)	N/A
Total paracenteses preceded by blood transfusion, n (%)				
Platelets	68 (5.3)	59 (9.7)	9 (1.3)	<0.001
Plasma	57 (4.4)	46 (7.6)	11 (1.6)	<0.001
Cryoprecipitate	4 (0.3)	0 (0)	4 (0.6)	0.05
	**Patient-Based Analysis**			
	**Total N = 449**	**Pre-TEG Protocol N = 253**	**Post-TEG Protocol N = 230**	***p***
Platelet count (x10^9^/L)	116 (74–169)	106 (72–158)	121 (78–179)	0.09
INR	1.6 (1.3–2.0)	1.6 (1.3–1.9)	1.6 (1.3–2.1)	0.26
TEG				
MA (mm)	—	—	48 (37–54)	N/A
R (min)	—	—	5 (4–6)	N/A
Total number of patients transfused with blood products, n (%)				
Platelets	28 (6.2)	22 (8.7)	6 (2.6)	0.004
Plasma	31 (6.9)	24 (9.5)	7 (3.0)	0.004
Cryoprecipitate	3 (0.7)	0 (0)	3 (1.3)	0.06

*Note:* Data are expressed as median (p25–p75) or proportions.

Abbreviations: MA, maximum amplitude; R, reaction time; TEG, thromboelastography.

### Onset of post-paracentesis hemoperitoneum

One patient developed PPH before the TEG-based protocol (0.4%) as did 1 patient after the TEG-based protocol cohort (0.4%). However, on further review of the post-TEG cohort patient’s course, it was determined that the episode of PPH was secondary to operator technique, causing an inadvertent arterial puncture based on direct visualization of extravasation from inferior epigastric artery seen on post-paracentesis abdominal imaging. Neither patient received pre-paracentesis blood product transfusions.

### Impact of TEG protocol on cost of care

The mean direct costs for these blood products, as well as the cost of performing TEG at our center during the study period, were obtained and compared based on product utilization rates between the 2 cohorts (Table 3). The overall decrease in blood product utilization following the implementation of the TEG protocol substantially outweighed the interval increase in TEG assay processing costs (pre-TEG cohort total costs: $50,082 vs. post-TEG cohort total costs: $12,585), resulting in a comparative cost saving of $37,497. The comparative cost saving per paracentesis procedure was $63.85. Cost-benefit and cost-effectiveness analyses were not performed, given such low incidence in the clinical outcome of interest (PPH).

**TABLE 3:** Quantity of blood products transfused and associated direct costs of pre- and post-protocol cohorts

	Blood product or TEG	Total # products transfused or TEG	Direct cost per transfusion or TEG, $	Total direct cost of transfusions and TEG, $	Total direct cost of transfusions and TEG per paracentesis, $
Pre-TEG protocol (N=607)	Platelets	90	443	39,870	65.68
	Fresh frozen plasma	138	74	10,212	16.82
	Cryoprecipitate	0	216	0	0
	TEG	0	22	0	0
	Total	—	—	50,082	82.50
Post-TEG protocol (N = 674)	Platelets	15	443	6645	9.85
	Fresh frozen plasma	26	74	1924	2.85
	Cryoprecipitate	8	216	1728	2.56
	TEG	104	22	2288	3.39
	Total	—	—	12,585	18.65

Abbreviation: TEG, thromboelastography.

## DISCUSSION

In our study, the implementation of a TEG-guided transfusion protocol resulted in a significant reduction in preprocedural blood product utilization compared to prior utilization rates based solely on conventional parameters (PLT, INR). Additionally, this decrease in prophylactic blood product transfusions was safely tolerated, with no increased incidence of PPH occurring in the post-TEG protocol cohort. The associated decrease in preprocedural blood transfusions following TEG-based transfusion protocol demonstrates strong potential for cost savings without compromising the quality of care. These findings substantiate the use of TEG as a safe and effective method for guiding prophylactic preprocedural blood product transfusions in patients with cirrhosis.

Accurately predicting periprocedural bleeding risk in patients with cirrhosis is challenging. Given the aforementioned limitations of INR and PLT to determine bleeding risk in these patients, current guidelines advise against prophylactic preprocedural blood product transfusions based on these conventional parameters.^16^ Despite such recommendations, abnormal PLT and INR levels are still often cited by providers as informing preprocedural blood product utilization. Of note, the INR and PLT thresholds stated in our TEG protocol algorithm (PLT < 20,000, INR ≥ 4) were based on consensus from an institutional multidisciplinary protocol review process, and the thresholds are more abnormal than standard definitions of secondary coagulopathy and thrombocytopenia used by others, including professional societies.^16–18^ Despite using these higher thresholds of conventional markers of presumed increased procedural bleeding risk, we observed a marked decrease in prophylactic blood product use among the post-TEG cohort with no associated increase in procedural bleeding complications. This supports that TEG was useful in estimating the risk of PPH in patients with cirrhosis despite abnormal conventional parameters (ie, PLT or INR) and was a better tool to inform appropriate blood product utilization in this patient population. These positive outcomes should prompt consideration for revising the “standard” conventional parameters historically driving prophylactic preprocedural transfusion strategies.

Furthermore, by virtue of using these higher thresholds to determine the need for TEG, it is imperative to note that TEG was required for only 104 of the entire 674 paracenteses (15%) performed in the post-TEG cohort. This suggests that educating providers on the interpretation of conventional lab parameters to determine periprocedural bleeding risk was a leading contributing factor to the observed decreased blood product utilization. The value of provider education on clinical determination of blood product utilization in patients with cirrhosis cannot be underestimated, especially when taking into account the increased morbidity and mortality posed by transfusion-associated complications (eg, transfusion-associated circulatory overload, exacerbation of portal hypertension) among patients with advanced liver disease.^19^ Our study demonstrates that such education can directly and safely reduce unnecessary prophylactic transfusions that can spare patients’ associated health risks and reduce health care spending.

Importantly, some patients with cirrhosis who are waitlisted for a liver transplant also require renal transplantation either simultaneously or after liver transplant. In such patients, it is critical to limit unnecessary blood transfusions to prevent allosensitization, which is associated with increased waitlist time, increased risk of graft rejection, and lower graft survival compared to nonsensitized patients.^20^ This highlights the additional clinical value of utilizing TEG to guide periprocedural prophylactic blood product use among patients with cirrhosis.

In contrast to conventional parameters of coagulopathy, viscoelastic testing such as TEG provides a more global assessment of hemostatic function, including clot formation, stability, fibrinogen levels, platelet function, and thrombin generation.^21,22^ Along with this comprehensive assessment, TEG also provides several other advantages, including its ability to provide rapid, real-time results that are amenable to guide blood transfusion strategies in a preprocedural setting.^23^ In a systematic review and cost-effectiveness analysis of the use of TEG in patients deemed high clinical bleeding risk (cardiac surgery, trauma, postpartum hemorrhage), Whiting et al demonstrated that TEG analysis resulted in less transfusion of blood products, reduced complications, decreased hospitalization costs, and a reduction in mortality.^24^ Our study similarly found that improved resource utilization produced noninferior clinical outcomes while achieving associated decreased costs of care, though formal survival and cost analyses were not performed.

It is worth noting that our study has some limitations. First, this is a single-center study, which can predispose to limited generalizability of our findings. However, our large total cohort size, coupled with the strong similarity of several clinical factors among the cohorts, provided adequate power to detect significant results between the 2 groups. Secondly, our designated follow-up time consisted of 48 hours after paracentesis and, therefore, could have theoretically limited detection of delayed PPH, which can occur several days after paracentesis. Thirdly, we did not assess the fibrinogen level, which can impact the TEG MA results that we used to determine the need for platelet transfusion. Cases receiving cryoprecipitates did so based on available fibrinogen levels, which were obtained at the discretion of the attending physician and not contemplated by our protocol. The multidisciplinary collaborative effort, including a thorough pertinent literature review, that was used to develop this protocol’s transfusion algorithm does lend itself to further protocol revision should future studies demonstrate the utility of pre-paracentesis fibrinogen levels or incorporation of κ time/α angle into decision-making to prevent PPH among patients with cirrhosis. Lastly, primarily due to some constraints in accessible cost data at the time of the study, our cost analysis was restricted to direct transfusion costs at our center to assess for cost differences between the pre-TEG and post-TEG cohorts. Formal cost-effective analysis, including sensitivity analysis, is needed to better determine the potential for cost savings with periprocedural prophylactic TEG-based transfusion protocol in this patient population.

In conclusion, the implementation of a pre-paracentesis TEG-based transfusion protocol for patients with cirrhosis successfully resulted in decreased blood product use with no increased incidence of PPH. TEG-based protocols appear to better inform providers about appropriate pre-paracentesis blood product use and can contribute to higher-value care, though further studies, including formal cost-effectiveness analysis, are warranted.

## Supplementary Material

SUPPLEMENTARY MATERIAL

## References

[1] Tripodi A Salerno F Chantarangkul V Clerici M Cazzaniga M Primignani M. Evidence of normal thrombin generation in cirrhosis despite abnormal conventional coagulation tests. Hepatology. 2005;41:553–8. 15726661 10.1002/hep.20569

[2] Tripodi A Primignani M Chantarangkul V Clerici M Dell'Era A Fabris F. Thrombin generation in patients with cirrhosis: The role of platelets. Hepatology. 2006;44:440–5. 16871542 10.1002/hep.21266

[3] Lisman T Bakhtiari K Pereboom ITA Hendriks HGD Meijers JCM Porte RJ. Normal to increased thrombin generation in patients undergoing liver transplantation despite prolonged conventional coagulation tests. J Hepatol. 2010;52:355–61. 20132999 10.1016/j.jhep.2009.12.001

[4] Andriulli A Tripodi A Angeli P Senzolo M Primignani M Giannini EG. Hemostatic balance in patients with liver cirrhosis: Report of a consensus conference. Digest Liver Dis. 2016;48:455–67. 10.1016/j.dld.2016.02.008 27012444

[5] Lisman T Caldwell SH Burroughs AK Northup PG Senzolo M Stravitz RT. Hemostasis and thrombosis in patients with liver disease: the ups and downs. J Hepatol. 2010;53:362–71. 20546962 10.1016/j.jhep.2010.01.042

[6] Salooja N Perry DJ. Thrombelastography. Blood Coagul Fibrinolysis. 2001;12:327–37. 11505075 10.1097/00001721-200107000-00001

[7] Rout G Shalimar Gunjan D Mahapatra SJ Kedia S Garg PK. Thromboelastography-guided Blood Product Transfusion in Cirrhosis Patients With Variceal Bleeding: A Randomized Controlled Trial. J Clinl Gastroenterol. 2020;54:255–62. 10.1097/MCG.0000000000001214 31008867

[8] Kumar M Ahmad J Maiwall R Choudhury A Bajpai M Mitra LG. Thromboelastography-Guided Blood Component Use in Patients With Cirrhosis With Nonvariceal Bleeding: A Randomized Controlled Trial. Hepatology. 2020;71:235–46. 31148204 10.1002/hep.30794

[9] De Pietri L Bianchini M Montalti R De Maria N Di Maira T Begliomini B. Thrombelastography-guided blood product use before invasive procedures in cirrhosis with severe coagulopathy: A randomized, controlled trial. Hepatology. 2016;63:566–573. 26340411 10.1002/hep.28148

[10] Garcia-Saenz-de-Sicilia M Al-Obaid L Hughes DL Duarte-Rojo A. Mastering Core Recommendations during HEPAtology ROUNDS in Patients with Advanced Chronic Liver Disease. Semin Liver Dis. 2022;42:341–361; PMID: 35764316. Publisher: Georg Thieme Verlag KG. 35764316 10.1055/a-1886-5909

[11] Shaydakov ME Sigmon DF Blebea J. Thromboelastography. 2022 Apr 14 ■ ■. StatPearls [Internet]. Treasure Island (FL): StatPearls Publishing; 2022.

[12] Garcia-Tsao G Parikh CR Viola A. Acute kidney injury in cirrhosis. Hepatology. 2008;48:2064–77. 19003880 10.1002/hep.22605

[13] Boks AL Brommer EJP Schalm SW Van Vliet HHDM. Hemostasis and fibrinolysis in severe liver failure and their relation to hemorrhage. Hepatology. 1986;6:79–86. 3943792 10.1002/hep.1840060115

[14] Shah NL Caldwell SH. Assessing the risk of bleeding and clotting in cirrhosis. Clin Liver Dis. 2016;7:26–8. 10.1002/cld.528 PMC6490249 31041022

[15] Soslau G Schwartz AB Putatunda B Conroy JD Parker J Abel RF. Desmopressin-induced improvement in bleeding times in chronic renal failure patients correlates with platelet serotonin uptake and ATP release. Am J The Med Sci. 1990;300:372–9. 10.1097/00000441-199012000-00006 2264575

[16] O’Shea RS Davitkov P Ko CW Rajasekhar A Su GL Sultan S. AGA Clinical Practice Guideline on the Management of Coagulation Disorders in Patients With Cirrhosis. Gastroenterology. 2021;161:1615–27. 34579936 10.1053/j.gastro.2021.08.015

[17] Patel IJ Rahim S Davidson JC Hanks SE Tam AL Walker TG. Society of Interventional Radiology Consensus Guidelines for the Periprocedural Management of Thrombotic and Bleeding Risk in Patients Undergoing Percutaneous Image-Guided Interventions-Part II: Recommendations: Endorsed by the Canadian Association for Interventional Radiology and the Cardiovascular and Interventional Radiological Society of Europe. J Vasc Interventl Radiol. 2019;30:1168–84. 10.1016/j.jvir.2019.04.017 31229333

[18] Grabau CM Crago SF Hoff LK Simon JA Melton CA Ott BJ. Performance standards for therapeutic abdominal paracentesis. Hepatology. 2004;40:484–8. 15368454 10.1002/hep.20317

[19] Piccin A Spizzo G Popovski MA Russo FP Armanaschi L Vecchiato C. Transfusion-associated circulatory overload in gastroenterology. Blood Transfus. 2021;19:197–204. 33000753 10.2450/2020.0025-20 PMC8092035

[20] Scornik JC Bromberg JS Norman DJ Bhanderi M Gitlin M Petersen J. An update on the impact of pre-transplant transfusions and allosensitization on time to renal transplant and on allograft survival. BMC Nephrol. 2013;14:217. 24107093 10.1186/1471-2369-14-217 PMC4125965

[21] Reikvam H Steien E Hauge B Liseth K Hagen KG Størkson R. Thrombelastography. Transfusion And Apheresis Sci. 2009;40:119–23. 10.1016/j.transci.2009.01.019 19249246

[22] Bolliger D Seeberger MD Tanaka KA. Principles and practice of thromboelastography in clinical coagulation management and transfusion practice. Transf Med Rev. 2012;26:1–13. 10.1016/j.tmrv.2011.07.005 21872428

[23] Shenoy A Louissaint J Shannon C Tapper EB Lok AS. Viscoelastic Testing Prior to Non-surgical Procedures Reduces Blood Product Use Without Increasing Bleeding Risk in Cirrhosis. Digest Dis Sci. 2022;67:5290–9. 35122595 10.1007/s10620-021-07376-6 PMC9352812

[24] Whiting P Al M Westwood M Ramos IC Ryder S Armstrong N. Viscoelastic point-of-care testing to assist with the diagnosis, management and monitoring of haemostasis: A systematic review and cost-effectiveness analysis. Health Technol Assess. 2015;19:1–228. 10.3310/hta19580 PMC4781169 26215747

