# Supplementary material for: TEG-based transfusion protocol is associated with decreased blood product use without increased risk of hemoperitoneum
Source: Hepatol Commun. 2023 Oct 27;7(11):e0292. doi: 10.1097/HC9.0000000000000292 (PMC10615392; doi:10.1097/HC9.0000000000000292)
Supplement: SUPPLEMENTARY MATERIAL [file hc9-7-e0292-s001.docx]

**Supplementary Table: Baseline demographic and clinical characteristics of entire cohort by Paracenteses**

|  |  | **Total**  **N=1281** | **Period 1**  **N=607** | **Period 2 N=674** | **p-value** |
| --- | --- | --- | --- | --- | --- |
| Age | Mean | 56 ±12 | 56 ±12 | 56 ±12 | 0.84 |
| Gender | Male | 656 (51%) | 335 (55%) | 321 (48%) | 0.007 |
|  | Female | 211 (44%) | 103 (41%) | 108 (47%) |  |
|  |  |  |  |  |  |
| Race | White | 1,172 (91%) | 558 (92%) | 614 (91%) | <0.001 |
|  | Black | 42 (3%) | 32 ( 5%) | 10 (1%) |  |
|  | Asian | 2 (0%) | 0 (0%) | 2 (1%) |  |
|  | Other | 63 (6%) | 17 (6%) | 48 (6%) |  |
|  |  |  |  |  |  |
| Etiologies of Cirrhosis | NASH | 386 (30%) | 175 (29%) | 211 (31%) | <0.001 |
|  | Alcohol | 539 (42%) | 243 (40%) | 296 (44%) |  |
|  | HCV | 160 (12%) | 93 (15%) | 67 (10%) |  |
|  | Other | 196 (16%) | 96 (16%) | 100 (15%) |  |
|  |  |  |  |  |  |
| Comorbidities | Diabetes Mellitus | 456 (36%) | 221 (36%) | 235 (35%) | 0.27 |
|  | Coronary artery disease | 178 (14%) | 46 (18%) | 35 (15%) | 0.38 |
|  | Chronic kidney disease | 441 (34%) | 69 (27%) | 64 (28%) | 0.89 |
|  | Dialysis | 125 (10%) | 24 (9%) | 16 (6%) | 0.11 |
|  |  |  |  |  |  |
| MELD-NA Score |  | 22 ± 5 | 21 ± 6 | 22 ± 5 | 0.20 |
|  |  |  |  |  |  |
| Cirrhosis-related complications | Hepatic Encephalopathy | 954 (74%) | 469 (77%) | 485 (72%) | 0.030 |
|  | Esophageal varices | 978 (76%) | 452 (74%) | 526 (78%) | 0.038 |
|  | Variceal bleeding | 263 (21%) | 114 (19%) | 149 (22%) | 0.14 |
|  | HCC | 89 (7%) | 37 (6%) | 52 (8%) | 0.33 |
| Aspirin |  | 236 (18%) | 93 (15%) | 67 (10%) | 0.015 |
| Low molecular weight heparin prophylaxis |  | 160 (12%) | 51 (20%) | 47 (20%) | 0.004 |
|  |  |  |  |  |  |
| Paracentesis setting | Inpatient | 732 (57%) | 345 (57%) | 387 (57%) | 0.83 |
|  | Outpatient | 549 (43%) | 262 (43%) | 287 (43%) |  |
